# Medicare spending and most-favored-nation pricing models

**DOI:** 10.1093/haschl/qxag254

**Published:** 2026-09-19

**Authors:** Matthew Vogel, Shiloh Liu, Vikram Sharma, Olivier J Wouters

**Affiliations:** John F. Kennedy School of Government, Harvard University, Cambridge, MA 02138, United States; John F. Kennedy School of Government, Harvard University, Cambridge, MA 02138, United States; Department of Health Services, Policy, and Practice, Brown University School of Public Health, Providence, RI 02903, United States; Department of Health Services, Policy, and Practice, Brown University School of Public Health, Providence, RI 02903, United States

**Keywords:** drug pricing, GLOBE, GUARD, international reference pricing, Medicare, most-favored-nation pricing, pharmaceutical policy, prescription drugs

Most-favored-nation (MFN) pricing is central to the Trump administration's prescription drug agenda. It aims to prevent drug companies from charging higher prices in the United States than in other developed countries. In December 2025, the Centers for Medicare and Medicaid Services proposed 2 mandatory MFN demonstration models: GLOBE for Medicare Part B and GUARD for Medicare Part D.^1,2^ Both models would require manufacturers to pay Medicare rebates when US prices exceed international benchmarks.

The administration has separately announced voluntary MFN agreements with 26 drug manufacturers under which participating companies provide MFN pricing in Medicaid, offer direct-to-consumer discounts, make launch-price commitments, and invest domestically.^3^ The agreements also appear to exempt participating manufacturers from other federal pricing initiatives, including GLOBE and GUARD. At least 10 manufacturers have publicly disclosed or confirmed exemptions from Medicare pricing demonstration models in securities filings, investor disclosures, earnings calls, and comments to the Centers for Medicare and Medicaid Services (Table S1). Some explicitly identify GLOBE and GUARD as covered exemptions. The administration has neither confirmed nor denied these exemptions but has explicitly reserved discretion to exempt manufacturers participating in other drug pricing initiatives.^1,2^

Despite concerns raised by commentators and legislators, the administration has released no estimate of the impact on the model-eligible spending base if the 26 manufacturers with voluntary agreements are exempted from GLOBE and GUARD.

In this study, we estimated the 2024 Medicare spending base on drugs meeting the proposed criteria for GLOBE and GUARD and quantified the share from products marketed by manufacturers with voluntary MFN agreements.

## Methods

We identified prescription drugs meeting the proposed GLOBE and GUARD criteria.^1,2^ In brief, we applied the models’ spending thresholds (>$100 million in annual spending in Part B or ≥$69 million in Part D) using the 2024 Part B and Part D Drug Spending Dashboards. Consistent with the proposals, we sequentially excluded products that were not Part B or Part D rebatable drugs under the Inflation Reduction Act; outside covered therapeutic areas; facing generic or biosimilar competition; or selected for Medicare drug price negotiation. Filters were applied at Healthcare Common Procedure Coding System (HCPCS) Level II for GLOBE and National Drug Code level for GUARD. Detailed methods are provided in the Supplementary material.

We classified products by application type (New Drug Application vs Biologics License Application) and route of administration using the US Food and Drug Administration (FDA) Orange and Purple Books. We assigned therapeutic categories using US Pharmacopeia classifications. We identified each product's manufacturer using FDA data and company disclosures.

We identified the scope of products and share of Medicare spending potentially subject to the proposed GLOBE and GUARD models. We separately estimated spending on products marketed by manufacturers with voluntary MFN agreements, treating this as an upper-bound estimate on the potential spending base excluded from GLOBE and GUARD if those products are exempted.

## Results

In 2024, 245 drugs met the proposed GLOBE or GUARD inclusion criteria (Figure 1). GLOBE drugs accounted for $28.5 billion, or 47% of Part B Dashboard spending ($60.8 billion). GUARD drugs accounted for $84.9 billion, or 29% of Part D Dashboard spending ($288.7 billion).

**Figure 1.**
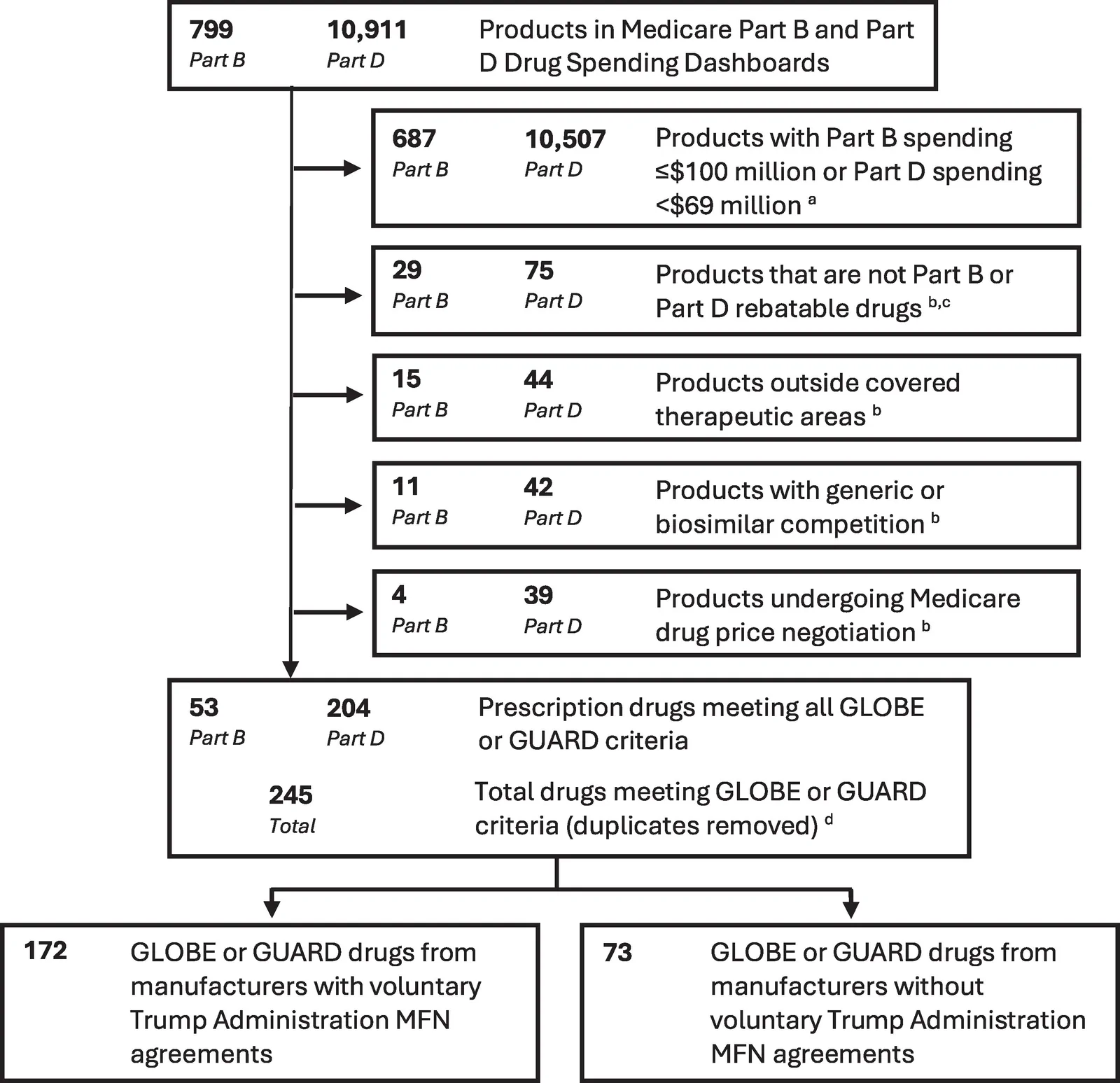
Identification of brand-name drugs meeting proposed criteria for Medicare most-favored-nation drug pricing. ^a^Under the proposed GLOBE rule, Medicare spending is aggregated at HCPCS Level II for purposes of applying a minimum annual threshold of $100 million in Part B fee-for-service drug spending. Under the proposed GUARD rule, spending is aggregated at the NDA or BLA level for purposes of applying a minimum annual threshold of at least $69 million in gross Part D drug spending across traditional Medicare and Medicare Advantage. For the products identified in this analysis, aggregating spending at HCPCS level II/NDC level was identical to aggregating spending at the NDA or BLA level. ^b^The proposed GLOBE rule applies all criteria at the HCPCS Level II level. The proposed GUARD rule applies all criteria (other than spending) at the NDC-9 level. For interpretability and comparability between Part B and Part D, we reported drug counts at the NDA/BLA level for both programs. ^c^Rebatable drug is a statutory category under the Medicare Inflation Rebate Program and generally refers to single-source products covered under Part B and Part D, subject to statutory and regulatory exclusions. ^d^Most drugs meeting the GLOBE or GUARD criteria qualified for only one of the programs. Twelve drugs met the criteria for both GUARD and GLOBE. Each drug was counted only once in the total. Abbreviations: BLA, Biologics License Application; HCPCS, Healthcare Common Procedure Coding System; MFN, most-favored-nation; NDA, New Drug Application; NDC, National Drug Code.

Spending was concentrated in antineoplastics ($34.8 billion; 31% of $113.4 billion), immunological agents ($16.8 billion; 15%), and blood glucose regulators ($13.2 billion; 12%). Biologics License Applications accounted for $52.6 billion (46%) and New Drug Applications $60.8 billion (54%). Oral products accounted for $43.3 billion (38%), injectable products $62.2 billion (55%), and products administered via other routes $7.9 billion (7%) (Table 1).

**Table 1.** Medicare spending in 2024 on brand-name drugs meeting proposed criteria for most-favored-nation drug pricing models.

		2024 Medicare spending in billions of $ (% from voluntary MFN agreement manufacturers)	
	No.	Part B/GLOBE^b^	Part D/GUARD^b^
All drugs^a^	245	28.5 (92%)	84.9 (74%)
Therapeutic category			
Analgesic	3	*	0.7 (0%)
Anticonvulsant	2	*	0.4 (0%)
Antidepressant	4	*	1.2 (10%)
Antigout	1	0.3 (100%)	**
Antimigraine	6	*	2.0 (100%)
Antineoplastic	77	18.0 (98%)	16.8 (62%)
Antipsychotic	7	*	3.9 (71%)
Antiviral	19	*	6.4 (98%)
Blood glucose regulator	20	*	13.2 (100%)
Blood product	4	1.5 (100%)	**
Cardiovascular	7	*	3.5 (85%)
Central nervous system	12	0.7 (100%)	3.9 (14%)
Gastrointestinal	7	*	1.1 (10%)
Genetic, enzyme, or protein disorder	12	*	6.6 (84%)
Immunological	29	4.4 (59%)	12.3 (83%)
Metabolic bone disease	2	0.6 (100%)	0.4 (27%)
Ophthalmic	14	3.0 (100%)	2.9 (42%)
Respiratory tract/pulmonary	19	*	9.5 (73%)
FDA application type			
BLA	92	27.1 (94%)	25.5 (84%)
NDA	153	1.4 (49%)	59.3 (69%)
Route of administration			
Oral	116	0.0 (not applicable)	43.3 (65%)
Injectable	105	28.5 (92%)	33.7 (87%)
Other^c^	24	0.0 (not applicable)	7.9 (64%)

Abbreviations: BLA, Biologics License Application; FDA, Food and Drug Administration; MFN, most-favored-nation; NDA, New Drug Application.

^a^Counts at the FDA application level.

^b^This table shows national spending totals. Totals may not equal the sum of components due to rounding. During the demonstration periods, GLOBE and GUARD would operate in selected geographic areas representing 25% of enrollees, which may or may not scale proportionally to national spending.

^c^Includes 10 inhaled, 9 ophthalmic, 2 topical, 2 nasal, and 1 buccal.

^*^Therapeutic area covered by GUARD, not GLOBE.

^**^Therapeutic area covered by GLOBE, not GUARD.

Among the products that met the inclusion criteria, 172 (70%) were marketed by manufacturers with voluntary MFN agreements. These products accounted for $26.3 billion (92%) of spending on Part B drugs meeting the proposed GLOBE criteria and $62.7 billion (74%) of spending on Part D drugs meeting the proposed GUARD criteria.

## Discussion

Among the drugs meeting the proposed GLOBE and GUARD inclusion criteria, products marketed by manufacturers with voluntary MFN agreements accounted for 92% of Part B spending and 74% of Part D spending. Exempting these products could therefore reduce potential Medicare savings. Similar concerns have been raised about the Inflation Reduction Act, where exemptions from the Medicare Drug Price Negotiation Program excluded large amounts of spending from reform efforts.^4,5^

Our analysis is illustrative, not a forecast, and has limitations. In June 2026, the Centers for Medicare and Medicaid Services submitted final versions of the GLOBE and GUARD rules for White House review, but the timing of their release is unknown. The final models may differ from the proposed rules or may not be finalized at all. As demonstration models, GLOBE and GUARD will only cover drug spending for 25% of beneficiaries. The most recent Medicare spending data available is from 2024 and may not reflect changes in utilization. Part D spending is reported gross of rebates. Because potential MFN rebates would represent a fraction of total spending, our estimates reflect spending potentially affected by GLOBE and GUARD rather than company rebate liability or the share of Medicare savings eliminated. Our estimates do not include spending on drugs whose eligibility for the models may change during the demonstration periods, for example, because spending rises above the minimum spending threshold or a drug becomes subject to a negotiated price under the Inflation Reduction Act.

The administration should clarify whether voluntary MFN agreements affect product inclusion in GLOBE and GUARD. Extensive exemptions for voluntary-agreement manufacturers could substantially reduce projected Medicare savings and weaken the evidentiary value of the demonstration programs.

## Supplementary Material

qxag254_Supplementary_Data
