# Supplementary material for: Medicare spending and most-favored-nation pricing models
Source: Health Aff Sch. 2026 Sep 19;4(10):qxag254. doi: 10.1093/haschl/qxag254 (PMC13663799; doi:10.1093/haschl/qxag254)
Supplement: Supplementary file 1 [file qxag254_supplementary_data.zip › supplement[2026.9.10].docx]

**Supplement**

Vogel M, Liu S, Sharma V, Wouters OJ. Medicare spending and most-favored-nation pricing models.

**Table S1.** Drug company disclosures of exemptions from Medicare MFN demonstration models.

**Supplementary Methods**. Additional information about study methods.

**Table S1.** Drug company disclosures of exemptions from Medicare MFN demonstration models.

| **Manufacturer** | **Disclosure** | **Source** |
| --- | --- | --- |
| **AbbVie** | Disclosed a 3-year exemption from “future price mandates.” Management subsequently stated that the exemption was “inclusive of demonstration projects.” | Form 10-Q filed with the SEC  April 28, 2026 ^[1]^  Q4 2025 earnings call  February 4, 2026 ^[2]^ |
| **BeOne** | A company spokesperson stated that its agreement “exempts the company’s medicines from two upcoming pilot programs testing price reductions in Medicare.” | Bloomberg Law  August 31, 2026 ^[3]^ |
| **BridgeBio** | Stated that it expected that it “will not be subject to future pricing mandates.” | Form 8-K (Exhibit 99.1) filed with the SEC  August 31, 2026 ^[4]^ |
| **Bristol Myers Squibb** | Stated that it “will not be subject to future pricing mandates in the US while the agreement remains in effect.” | Form 10-Q filed with the SEC  April 21, 2026 ^[5]^ |
| **Eli Lilly** | Stated that, because of its agreement with the Trump administration, it “will not be subject to future pricing mandates.” | Investor release  November 6, 2025 ^[6]^ |
| **Genentech** | Stated that it would not be subject to future pricing mandates. | Genentech press release December 19, 2025 ^[7]^ |
| **Gilead** | Confirmed to Wall Street analysts that it would not be subject to future pricing mandates. In comments on GLOBE and GUARD, stated that companies participating in GENEROUS are exempt and urged CMS to make the exemption explicit in the final rules. | Jefferies analyst report  December 20, 2025 ^[8]^  Comments to CMS  February 23, 2026 ^[9],^^[10]^ |
| **Merck** | Confirmed to Wall Street analysts that under its MFN agreement it would not be subject to future pricing mandates. | Leerink analyst report  December 21, 2025 ^[11]^ |
| **Pfizer** | Chief executive officer stated that the agreement provided “clarity on pricing framework” and “removed uncertainty regarding tariffs and drug pricing.” | Pfizer press release  September 30, 2025 ^[12]^  Leerink analyst report  September 30, 2025 ^[13]^ |
| **Regeneron** | Stated that following its voluntary MFN agreement it “will not be subject to future US government pricing mandates.” | Q1 2026 earnings release  April 29, 2026 ^[14]^ |
| **Roche *** | Stated that, as part of its agreement with the US government, its portfolio is “exempt from tariffs and demo projects (GUARD and GLOBE).” | Roche investor presentation  January 29, 2026 ^[15]^ |
| **UCB** | Stated that it will not be subject to “future pricing mandates.” | Press release  August 31, 2026 ^[16]^ |

* Roche is the parent company of Genentech.

CMS indicates Centers for Medicare and Medicaid Services; MFN, most-favored-nation; SEC, Securities and Exchange Commission.

**Supplementary Methods.** Additional information about study methods.

**Background**

The Centers for Medicare and Medicaid Services (CMS) proposed the Global Benchmark for Efficient Drug Pricing (GLOBE) and Guarding U.S. Medicare Against Rising Drug Costs (GUARD) demonstration models, both of which would be mandatory for all manufacturers selling qualifying medicines to Medicare beneficiaries. As demonstration models, GLOBE would include 25% of Medicare Part B fee-for-service beneficiaries and GUARD would include 25% of beneficiaries enrolled in either a stand-alone or a Medicare Advantage Part D prescription drug plan.

We conducted a cross-sectional analysis applying the inclusion criteria specified in the proposed GLOBE and GUARD models. Because both models remain proposed rather than final, our estimates should be interpreted as an application of the proposed rule to currently available public data. CMS advanced the final GUARD rule to the White House on June 15, 2026,^[17]^ and the final GLOBE rule on June 26, 2026.^[18]^

All analyses were conducted in Excel (version 16; Microsoft). Because this study used public, aggregate data and did not involve patient-level information, institutional review board review was not required. We followed the Strengthening the Reporting of Observational Studies in Epidemiology (STROBE) reporting guideline.

**Public Information Regarding Manufacturer Exemptions From GLOBE and GUARD**

The possible exemption of manufacturers from GLOBE and GUARD due to their participation in voluntary MFN agreements has been raised repeatedly by congressional, industry, and other stakeholders.

On March 5, 2026, the ranking Democrats on the Senate Finance Committee, House Energy and Commerce Committee, and House Ways and Means Committee wrote to the Secretary of Health and Human Services, Robert F. Kennedy Jr., stating that “pharmaceutical companies that have reached drug pricing agreements with the White House are openly telling the press and their shareholders that they are exempt from GLOBE and GUARD. In other words, manufacturers may be able to get out of providing international level drug prices in Medicare by agreeing to separate drug pricing deals with the White House.”^[19]^ The members called on the administration to make available to Congress the full text of the agreements, including “any language or terms that could result in manufacturer exemptions from the GLOBE and GUARD Models.”

Individual company disclosures have ranged from vague assurances about future drug pricing initiatives to detailed statements in filings with the US Securities and Exchange Commission, where materially false or misleading statements expose companies to federal civil and criminal liability.

Ahead of the most recent (August 31, 2026) White House announcement of manufacturer participation in the voluntary GENEROUS Medicaid model, Bloomberg reported that “the companies will be exempt from pilot programs forcing similar discounts in Medicare.”^[20]^

Concern about exemptions for manufacturers entering voluntary agreements was also a theme in public comments during the GLOBE and GUARD rulemaking process, including in comments to CMS submitted by the American Association of Retired Persons, Arnold Ventures, Association for Clinical Oncology, Brookings Institution, Families USA, National Association of Specialty Pharmacy, Patients for Affordable Drugs, Pharmaceutical Care Management Association, Public Citizen, Teva Pharmaceuticals, and UnitedHealth Group.^[21],^^[22]^

**Identification of Drugs Meeting Proposed GLOBE or GUARD Inclusion Criteria**

We identified drugs potentially subject to the proposed GLOBE and GUARD models by applying the proposed model criteria as sequential filters: (1) exclusion of products with Medicare spending below the model-specific threshold, (2) exclusion of products that were not Part B or Part D rebatable prescription drugs, (3) exclusion of products outside of covered therapeutic categories, (4) exclusion of reference products with marketed generic or biosimilar competition, and (5) exclusion of products under the Inflation Reduction Act’s Medicare Drug Price Negotiation Program.

Exclusion filters were applied as specified in the proposed rules: Healthcare Common Procedure Coding System (HCPCS) Level II billing codes for GLOBE and National Drug Code level for GUARD. We obtained NDC information from the Food and Drug Administration (FDA) NDC Directory.^[23]^ The sole exception is that the spending threshold was applied at the New Drug Application (NDA) or Biologics License Application (BLA) level for GUARD, as specified in the proposed rule.

1. Exclusion of products with Medicare spending below the model-specific threshold

Consistent with prior research evaluating the potential impact of Medicare drug pricing reforms, spending was measured as total spending reported in the Medicare Drug Spending Dashboards.^[24],^^[25],^^[26],^^[27],^^[28],^^[29],^^[30],^^[31]^ We used the most recently updated version of the Part B and Part D dashboards, which covered calendar year 2024 (released on July 9, 2026), to apply the spending thresholds specified in the proposed GLOBE and GUARD rules. We did not inflate or project 2024 spending to the model implementation years.

Medicare Part B spending was measured using the Medicare Part B Drug Spending Dashboard, which summarizes traditional Medicare fee-for-service claims at the HCPCS Level II and reports spending as the full value of the product, including Medicare payment and beneficiary liability. Medicare Part D spending was measured using the Medicare Part D Drug Spending Dashboard, which summarizes spending in stand-alone prescription drug plans and Medicare Advantage prescription drug plans. Part D spending reflects gross drug costs, including payments by Medicare, stand-alone plans, and beneficiaries, and does not reflect manufacturer rebates or other post-sale concessions.

For GLOBE, the proposed rule identifies eligible Part B drugs by HCPCS Level II code. Consistent with the proposed rule, we include only Part B drugs with more than $100 million in annual Medicare Part B fee-for-service spending per HCPCS Level II code.^[32]^

For GUARD, the proposed rule identifies drugs at the NDC-9 level but applies the minimum Part D spending threshold using application-level total gross covered prescription drug costs. Application-level total gross covered prescription drug costs are defined in the proposed rule as the sum of gross covered prescription drug costs in Medicare Part D for all rebatable Part D drugs belonging to the same FDA application. Consistent with the proposed rule, only Part D drugs with at least $69 million in annual gross Medicare Part D spending, aggregated at the NDA or BLA level, were included.^[33]^

2. Exclusion of products that are not Part B or Part D rebatable prescription drugs

We classified products as brand-name prescription drugs if they were marketed under an FDA-approved NDA or BLA and were not generic or biosimilar products. We excluded products identified as abbreviated new drug application products, biosimilars, or products otherwise marketed as non-originator versions. We also excluded products that were included in the Medicare Drug Spending Dashboard files but that were medical devices, such as needles and insulin pumps, or which are categorically excluded under the proposed rules. For example, GLOBE excludes products including skin substitutes, certain vaccines and related products (influenza, pneumococcal, hepatitis B, and COVID-19 vaccines and antibodies used for the post-exposure prophylaxis of COVID-19), and radiopharmaceuticals.

3. Exclusion of products outside of covered therapeutic categories

As specified in the proposed rules, a product was treated as meeting the therapeutic-category criterion if at least one assigned US Pharmacopeia classification was included in the model’s selected categories, even if a product had other classifications not covered by the models.

The proposed GLOBE rule, based on the US Pharmacopeia Drug Classification system,^[34]^ covers the following categories: antigout agents, antineoplastics, blood products and modifiers, central nervous system agents, immunological agents, metabolic bone disease agents, and ophthalmic agents.

The proposed GUARD rule, based on the US Pharmacopeia Medicare Model Guidelines,^[35]^ covers the following categories: analgesics, anticonvulsants, antidepressants, antimigraine agents, antineoplastics, antipsychotics, antivirals, bipolar agents, blood glucose regulators, cardiovascular agents, central nervous system agents, gastrointestinal agents, genetic or enzyme or protein disorder treatments, immunological agents, metabolic bone disease agents, ophthalmic agents, and respiratory tract or pulmonary agents.

We included all products in covered therapeutic categories. This included products that were exempted from the Medicare Drug Price Negotiation Program under the Inflation Reduction Act, such as treatments derived from human plasma. We also included cell and gene therapies because they are covered in the proposed rules. However, we note that the administration has indicated that it is open to potentially exempting these products.

4. Exclusion of reference products facing competition from marketed generic or biosimilar versions

We excluded brand-name drugs that are reference products with therapeutic-equivalent generic competition or biosimilar competition. Approved generic and biosimilar products were identified using the FDA’s NDC database, Orange Book, and Purple Book. We determined that generic or biosimilar entry had occurred once a product had a marketing start date in the Medicaid Prescription Drug Rebate Program database.^[36]^ Authorized generics and unbranded biologics marketed under the originator NDA or BLA were treated as part of the originator product.

5. Exclusion of products under the Inflation Reduction Act’s Medicare Drug Price Negotiation Program

GLOBE and GUARD exclude products with maximum fair prices (i.e., negotiated prices) in effect under the Inflation Reduction Act’s Medicare Drug Price Negotiation Program. We excluded all drugs selected for Medicare price negotiation as of September 9, 2026, including those with maximum fair prices not yet in effect. We applied this exclusion based on CMS-published lists of drugs selected for Medicare price negotiation.^[37]^ This approach removes drugs already known to become ineligible during the proposed model periods but does not account for drugs that may be selected in future negotiation cycles.

**Identification of Products Marketed by Manufacturers Participating in Voluntary MFN Agreements with the Trump Administration**

As of September 9, 2026, the White House or manufacturers had announced voluntary MFN agreements between the Trump administration and the following 26 manufacturers: AbbVie, Alcon, Amgen, Astellas, AstraZeneca, BeOne, Boehringer Ingelheim, BridgeBio, Bristol Myers Squibb, CSL, Eli Lilly, EMD Serono, Genentech, Gilead, GlaxoSmithKline, Johnson & Johnson, Kyowa Kirin, Merck, Novartis, Novo Nordisk, Pfizer, Regeneron, Sanofi, Sun, Teva, and UCB.^[38]^ Manufacturer participation in voluntary MFN agreements was determined based on press releases from the White House and manufacturers.

We identified the manufacturer(s) of each product according to a prespecified hierarchy. First, we identified the FDA application holder using the Orange Book for NDAs and the Purple Book for BLAs. Second, we identified labelers using the FDA’s NDC Directory.^[39]^ Third, we reviewed public company sources, including product websites, prescribing-information pages, press releases, and SEC filings. When these sources differed, we assigned the product to the company identified in public materials as having US commercial responsibility for the product. Product-level manufacturer assignments were manually reviewed by two study team members (M.V. and S.L.), with discrepancies resolved by consensus.

For co-marketed products, joint ventures, or products with multiple manufacturers, we classified the product as associated with a voluntary-agreement manufacturer if a manufacturer with US marketing responsibility had entered a voluntary MFN agreement.

**Drug Characteristics Coding**

We classified drugs by (1) therapeutic category, (2) application type, and (3) route of administration. Therapeutic category was assigned using US Pharmacopeia categories. To determine the application type (NDA vs. BLA) and route of administration (oral vs injectable vs other) for each product, we used the FDA’s Orange Book (“Approved Drug Products with Therapeutic Equivalence Evaluations”)^[40]^ and Purple Book (“Database of Licensed Biological Products”).^[41]^ Routes of administration were grouped as oral, injectable, or other. Oral products included tablets, capsules, and other solid-dose formulations. Injectable products included intravenous, subcutaneous, intramuscular, intraocular, intravitreal, and other parenteral routes. Other routes included inhaled, topical, ophthalmic, intranasal, transdermal, rectal, and vaginal. For products available in multiple formulations, route was assigned based on the formulation associated with the highest level of Medicare spending where data was available; otherwise, the lead marketed formulation was used.

**Product Counts and Spending Reporting**

We reported product counts and spending at the FDA application (i.e., NDA or BLA) level. We chose this level of aggregation because we found that it most closely tracks (though is not synonymous with) the HCPCS-level coding under GLOBE, the product lines in the drug spending dashboards, and the spending aggregation level under GLOBE and GUARD. Though the NDC-9 level will be used in GUARD to identify international analogs to calculate MFN benchmarks, we do not believe it is the most intuitive unit of analysis for reporting in this study and does not allow for comparisons across Part B and Part D. For the products identified in this study, aggregating spending at HCPCS level II/NDC level was identical to aggregating spending at the FDA application level.

We reported spending in nominal 2024 US dollars. Percentages were calculated using total 2024 Medicare Part B and Part D drug spending from the Drug Spending Dashboards as the denominator. For drugs with spending in both Part B and Part D, Part B spending was only included if the product met proposed GLOBE criteria and Part D spending was only included if the product met proposed GUARD criteria. Spending was reported at the FDA application level (i.e., NDA or BLA).

We quantified 2024 Medicare spending on products meeting proposed GLOBE or GUARD screening criteria and, separately, the subset marketed by manufacturers with publicly announced voluntary MFN agreements. Because the voluntary agreements are not public and CMS has not confirmed whether or how such agreements would affect GLOBE or GUARD participation, these estimates should be interpreted as spending associated with voluntary-agreement manufacturers, not as spending definitively excluded from the models or directly subject to MFN rebates.

**SUPPLEMENTAL REFERENCES**

1. AbbVie Q1 2026 Form 10-Q. April 28, 2026. Accessed: June 23, 2026. https://www.sec.gov/Archives/edgar/data/1551152/000155115226000017/abbv-20260331.htm
2. AbbVie Q4 2025 Earnings Call. February 4, 2026. Accessed: June 23, 2026. https://www.fool.com/earnings/call-transcripts/2026/02/04/abbvie-abbv-q4-2025-earnings-call-transcript/
3. Cohrs Zhang R, Woodhouse S, Phengsitthy N. Trump Adds Nine Drugmakers to Medicaid Price Deal Program. Bloomberg Law. August 31, 2026. Accessed September 9, 2026. https://news.bloomberglaw.com/social-justice/trump-strikes-medicaid-drug-price-deals-with-nine-pharma-firms
4. BridgeBio Pharma. BridgeBio Announces Agreement with U.S. Government to Improve Affordability and Access to Critical Medicines for Americans. Exhibit 99.1 to Current Report on Form 8-K. US Securities and Exchange Commission. August 31, 2026. Accessed: September 9, 2026. https://www.sec.gov/Archives/edgar/data/1743881/000114036126035055/ef20081330_ex99-1.htm
5. Bristol Myers Squibb 10-Q. April 21, 2026. Accessed: June 23, 2026. https://www.sec.gov/Archives/edgar/data/14272/000001427226000010/bmy-20260331.htm
6. Eli Lilly press release. November 6, 2025. Accessed: June 23, 2026. https://investor.lilly.com/node/53251/pdf
7. Genentech MFN press release. December 19, 2025. Accessed: June 23, 2026. https://www.gene.com/media/press-releases/15094/2025-12-19/genentech-announces-agreement-with-us-go
8. Jefferies. Thoughts on White House MFN Deals + GLOBE/GUARD Models. December 20, 2025.
9. Gilead comments on GLOBE. February 23, 2026. Accessed: June 23, 2026. https://www.regulations.gov/comment/CMS-2025-1889-0131
10. Gilead comments on GUARD. February 23, 2026. Accessed: June 23, 2026. https://www.regulations.gov/comment/CMS-2025-1888-0161
11. Leerink. Additional MFN Agreements Put Industry on Trump’s Good Side. December 21, 2025.
12. Pfizer press release. September 30, 2025. Accessed: June 23, 2026. https://www.pfizer.com/news/press-release/press-release-detail/pfizer-reaches-landmark-agreement-us-government-lower-drug
13. Leerink. PFE’s Voluntary Agreement Yields Relief; Note Limited PFE Medicaid Exposure. September 30, 2025.
14. Regeneron Q1 2026 earnings release, Exhibit 99.1. April 29, 2026. Accessed: June 23, 2026. https://www.sec.gov/Archives/edgar/data/872589/000087258926000014/exhibit991q12026.htm
15. Roche Annual Investor Presentation. January 29, 2026. Accessed: June 23, 2026. https://assets.roche.com/f/176343/x/dea78e334b/irp260129.pdf
16. UCB. UCB Comes to Agreement with U.S. Government to Lower the Cost of Medicine and Strengthen U.S. Manufacturing. August 31, 2026. Accessed: September 9, 2026. https://www.ucb.com/newsroom/press-releases/article/ucb-comes-to-agreement-with-us-government-to-lower-the-cost-of-medicine-and-strengthen-us-manufacturing
17. Office of Information and Regulatory Affairs, Office of Management and Budget. RIN: 0938-AV74. Accessed July 9, 2026. https://www.reginfo.gov/public/do/eoDetails?rrid=1429713.
18. Office of Information and Regulatory Affairs, Office of Management and Budget. RIN: 0938-AV66. Accessed July 9, 2026. https://www.reginfo.gov/public/do/eoDetails?rrid=1444715.
19. Letter to Secretary of Health and Human Services Kennedy from Democratic Members of Congress. March 5, 2026. Accessed: June 23, 2026. https://democrats-waysandmeans.house.gov/sites/evo-subsites/democrats-waysandmeans.house.gov/files/evo-media-document/hhs.2026.03.05.letter-re-globe-and-guard-models.pdf
20. Phengsitthy N, Cohrs Zhang R, Muller M. White House to announce new drug pricing deals with biotechs. Bloomberg Law. August 26, 2026. Accessed: September 9, 2026. https://news.bloomberglaw.com/insurance/white-house-to-announce-new-drug-pricing-deals-with-biotechs
21. Centers for Medicare & Medicaid Services. Global Benchmark for Efficient Drug Pricing (GLOBE) Model. 90 Fed Reg 60244. December 23, 2025. Accessed: May 23, 2026. https://www.cms.gov/priorities/innovation/innovation-models/globe.
22. Centers for Medicare & Medicaid Services. Guarding U.S. Medicare Against Rising Drug Costs (GUARD) Model. 90 Fed Reg 60338. December 23, 2025. Accessed: May 23, 2026. https://www.cms.gov/priorities/innovation/innovation-models/guard.
23. FDA National Drug Code Directory. Accessed: June 23, 2026. https://www.fda.gov/drugs/drug-approvals-and-databases/national-drug-code-directory
24. Vogel M, Zhao O, Feldman WB, Chandra A, Kesselheim AS, Rome BN. Cost of Exempting Sole Orphan Drugs From Medicare Negotiation. JAMA Intern Med. 2024 Jan 1;184(1):63-69.
25. Reitsma MB, Dusetzina SB, Ballreich JM, Trujillo AJ, Mello MM. Examining Opportunities to Increase Savings From Medicare Price Negotiations. JAMA Intern Med. 2023 Jun 1;183(6):581-588.
26. Rome BN, Nagar S, Egilman AC, Wang J, Feldman WB, Kesselheim AS. Simulated Medicare Drug Price Negotiation Under the Inflation Reduction Act of 2022. JAMA Health Forum. 2023;4(1):e225218.
27. Reitsma MB, Dusetzina SB, Ballreich JM, Trujillo AJ, Mello MM. Estimated Savings From Extending Prescription Drug Inflationary Rebates To All Commercial Plans. Health Aff (Millwood). 2025 Mar;44(3):256-264.
28. Qureshi O, Ramachandran R, Ross JS. Medicare Part B and Part D drug eligibility for center for Medicare and Medicaid Services price negotiation under the Inflation Reduction Act: estimates using 2016-2019 data. J Pharm Policy Pract. 2024 Feb 29;17(1):2312374.
29. Vogel M, Kakani P, Chandra A, Conti RM. Medicare price negotiation and pharmaceutical innovation following the Inflation Reduction Act. Nat Biotechnol. 2024 Mar;42(3):406-412.
30. Hernandez I, Gabriel N, Dickson S. Nonfederal average manufacturer price to estimate savings generated by minimum discounts under the Inflation Reduction Act. J Manag Care Spec Pharm. 2023 Nov;29(11):1261-1263.
31. Hwang TJ, Jain N, Lauffenburger JC, Vokinger KN, Kesselheim AS. Analysis of Proposed Medicare Part B to Part D Shift With Associated Changes in Total Spending and Patient Cost-Sharing for Prescription Drugs. JAMA Intern Med. 2019 Mar 1;179(3):374-380.
32. Centers for Medicare & Medicaid Services. Global Benchmark for Efficient Drug Pricing (GLOBE) Model. 90 Fed Reg 60244. December 23, 2025. Accessed: May 23, 2026. https://www.cms.gov/priorities/innovation/innovation-models/globe.
33. Centers for Medicare & Medicaid Services. Guarding U.S. Medicare Against Rising Drug Costs (GUARD) Model. 90 Fed Reg 60338. December 23, 2025. Accessed: May 23, 2026. https://www.cms.gov/priorities/innovation/innovation-models/guard.
34. United States Pharmacopeial Convention. USP Drug Classification 2025. US Pharmacopeia. Published January 29, 2026. Accessed May 23, 2026. <https://pages.usp.org/USPDC2025.html>.
35. United States Pharmacopeial Convention. USP Medicare Model Guidelines, Version 9.0. US Pharmacopeia. Accessed May 23, 2026. <https://www.usp.org/health-quality-safety/usp-medicare-model-guidelines>.
36. Centers for Medicare & Medicaid Services. Drug products in the Medicaid Drug Rebate Program. Accessed May 23, 2026. <https://data.medicaid.gov/dataset/0ad65fe5-3ad3-5d79-a3f9-7893ded7963a>.
37. Centers for Medicare & Medicaid Services. Selected Drugs and Negotiated Prices. Accessed: May 23, 2026. <https://www.cms.gov/initiatives/medicare-prescription-drug-affordability/overview/medicare-drug-price-negotiation-program/selected-drugs-negotiated-prices>.
38. The White House. Fact Sheet: President Donald J. Trump Announces Deal with Nine Additional Pharmaceutical Manufacturers to Lower Drug Prices for Americans. August 31, 2026. Accessed: September 9, 2026. https://www.whitehouse.gov/fact-sheets/2026/08/fact-sheet-president-donald-j-trump-announces-deal-with-nine-additional-pharmaceutical-manufacturers-to-lower-drug-prices-for-americans/
39. FDA National Drug Code Directory. Accessed: June 23, 2026. https://www.fda.gov/drugs/drug-approvals-and-databases/national-drug-code-directory
40. US Food and Drug Administration. Approved Drug Products With Therapeutic Equivalence Evaluations. Accessed: May 23, 2026. <https://www.fda.gov/drugs/drug-approvals-and-databases/approved-drug-products-therapeutic-equivalence-evaluations-orange-book>.
41. US Food and Drug Administration. Database of Licensed Biological Products. Accessed: May 23, 2026. [https://purplebooksearch.fda.gov](https://purplebooksearch.fda.gov/).
